# Sacubitril/Valsartana versus Enalapril ou Losartana em Doses Máximas na Insuficiência Cardíaca com Fração de Ejeção Reduzida: Dados de Mundo Real

**DOI:** 10.36660/abc.20260069

**Published:** 2026-07-30

**Authors:** Renato de Carvalho Barros, Vinícius C. Fiusa, Gabrielle N. Tim, Victoria T. V. Goulart, Caio Henrique M. Nascimento, Gustavo T. de Moura, Adriana J. B. A. Guimarães, Sergio Henrique Rodolpho Ramalho, Armindo Jreige, Filipe A. Moura, Luiz Sérgio Fernandes de Carvalho

**Affiliations:** 1 Universidade de Brasília Faculdade de Ciências Médicas Brasília DF Brasil Universidade de Brasília - Faculdade de Ciências Médicas, Brasília, DF – Brasil; 2 Laboratório de Dados para Qualidade do Cuidado e Pesquisa de Desfechos Brasília DF Brasil Laboratório de Dados para Qualidade do Cuidado e Pesquisa de Desfechos (LaDaQCOR), Brasília, DF – Brasil; 3 Escola Superior de Ciências da Saúde Grupo de Pesquisa em Doenças Cardiovasculares de Brasília Brasília DF Brasil Grupo de Pesquisa em Doenças Cardiovasculares de Brasília – Escola Superior de Ciências da Saúde (ESCS), Brasília, DF – Brasil; 4 Escola Superior de Ciências da Saúde Brasília DF Brasil Escola Superior de Ciências da Saúde (ESCS), Brasília, DF – Brasil; 5 Instituto Sírio-libanês de Ensino e Pesquisa São Paulo SP Brasil Instituto Sírio-libanês de Ensino e Pesquisa, São Paulo, SP – Brasil; 6 Instituto de Cardiologia do Distrito Federal Brasília DF Brasil Instituto de Cardiologia do Distrito Federal (ICDF), Brasília, DF – Brasil; 7 Yale School of Medicine Section of Cardiovascular Medicine New Haven EUA EUA Yale School of Medicine - Section of Cardiovascular Medicine, New Haven – EUA; 8 VA Connecticut Healthcare System West Haven EUA VA Connecticut Healthcare System, West Haven – EUA; 9 Universidade Católica de Brasília Taguatinga DF Brasil Universidade Católica de Brasília – Gerontologia, Taguatinga, DF – Brasil

**Keywords:** Insuficiência Cardíaca Sistólica, Valsartana, Enalapril

## Abstract

**Fundamento:**

Sacubitril/valsartana reduziu a mortalidade e as hospitalizações em pacientes com insuficiência cardíaca com fração de ejeção reduzida (ICFEr) em comparação com enalapril em estudos clínicos pivotais. No entanto, os dados de mundo real sob doses otimizadas continuam limitados.

**Objetivo:**

Comparar os desfechos de pacientes com ICFEr tratados com sacubitril/valsartana versus enalapril ou losartana nas doses máximas recomendadas pelas diretrizes.

**Métodos:**

Analisamos dados de uma coorte observacional de insuficiência cardíaca (n = 2.314), incluindo pacientes atendidos entre agosto de 2010 e setembro de 2024 em seis hospitais públicos de uma grande área metropolitana. A análise primária incluiu 254 pacientes com ICFEr que receberam as doses máximas recomendadas pelas diretrizes dos medicamentos investigados. Uma análise de sensibilidade incluiu 452 pacientes que receberam quaisquer doses submáximas. O desfecho primário foi a combinação de hospitalização ou óbito. As diferenças basais foram ajustadas por meio do pareamento por escore de propensão. Foram considerados significativos valores de p < 0,05.

**Resultados:**

Entre 254 pacientes com ICFEr (idade média de 63,6 ± 12,3 anos, 61,4% do sexo masculino), 115 receberam sacubitril/valsartana 97/103 mg duas vezes ao dia, 33 enalapril 20 mg duas vezes ao dia e 106 losartana 100 mg ao dia. Durante um seguimento mediano de 23,9 meses (intervalo interquartil 11,9–41,1), ocorreram 70 eventos do desfecho primário (56 primeiras hospitalizações e 32 óbitos). Em comparação com doses equivalentes de enalapril ou losartana, sacubitril/valsartana não foi associado a reduções significativas na combinação de hospitalização ou mortalidade (*odds ratio* 0,650, intervalo de confiança de 95% 0,354–1,195, p = 0,165), mas foi associado à melhora do estado funcional (*odds ratio* 3,902, intervalo de confiança de 95% 1,745–8,726, p = 0,001).

**Conclusão:**

O presente estudo de mundo real não demonstrou reduções significativas de hospitalização ou mortalidade com sacubitril/valsartana em comparação a enalapril ou losartana nas doses máximas recomendadas pelas diretrizes; no entanto, sacubitril/valsartana foi associado à melhora da classe funcional.

**Figure:**
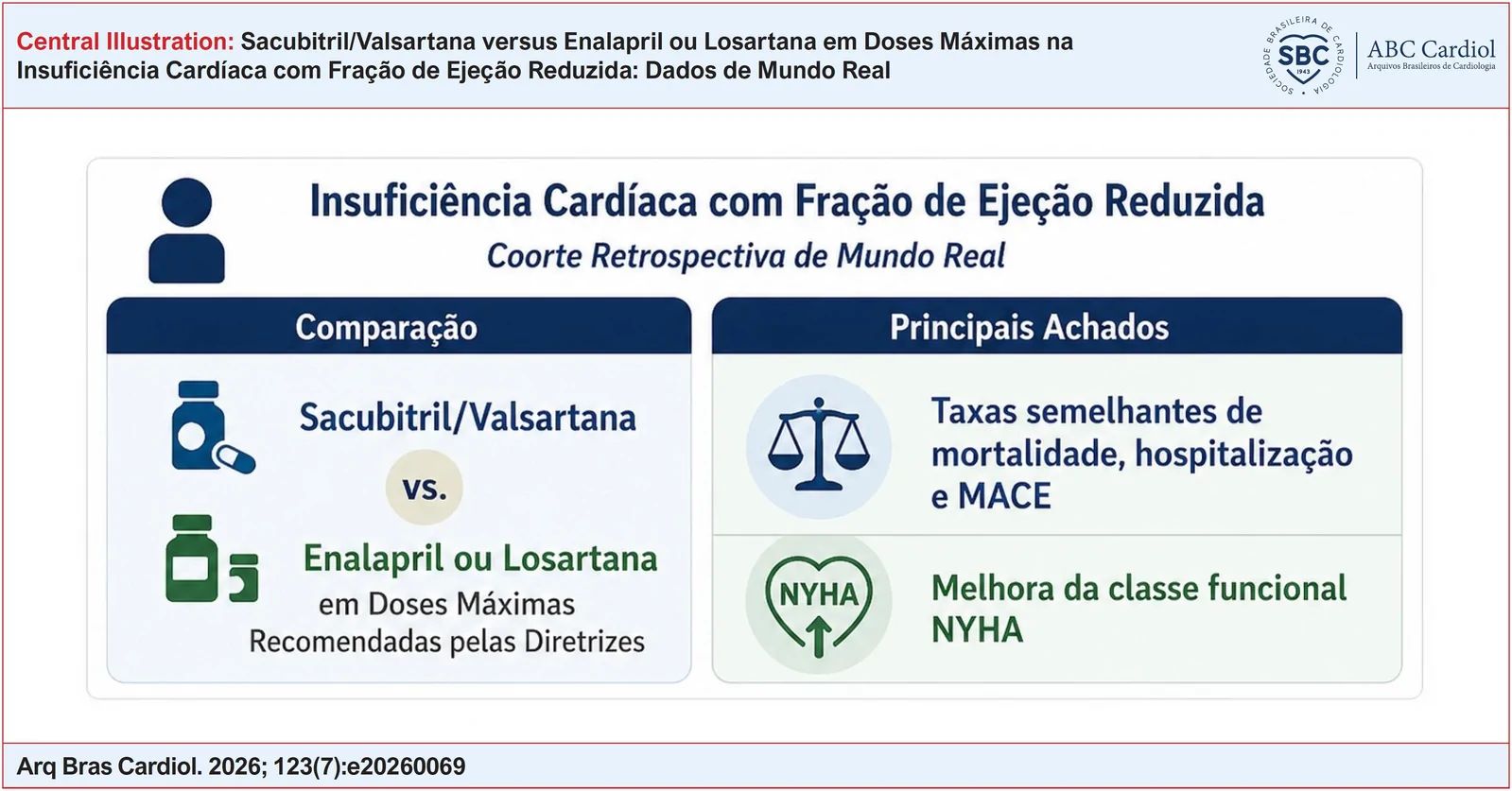
MACE: eventos cardiovasculares adversos maiores; NYHA: New York Heart Association.

## Introdução

Na última década, os inibidores do receptor de angiotensina-neprilisina (ARNI) surgiram como tratamento fundamental para a insuficiência cardíaca com fração de ejeção reduzida (ICFEr). A combinação de sacubitril com valsartana promove vasodilatação e natriurese, ao mesmo tempo que neutraliza os efeitos cardiotóxicos da angiotensina II.^1^

As diretrizes atuais para insuficiência cardíaca recomendam os ARNI em vez dos inibidores da enzima conversora de angiotensina (IECA) ou dos bloqueadores dos receptores de angiotensina (BRA) para pacientes com ICFEr.^2,3^ Essa recomendação foi inicialmente embasada pelo estudo PARADIGM-HF, que demonstrou uma redução significativa na mortalidade e nas hospitalizações com sacubitril/valsartana em comparação com enalapril.^4^ Posteriormente, uma análise conjunta de dados dos ensaios PIONEER-HF e PARAGLIDE-HF forneceu evidências adicionais em favor do uso de ARNI na insuficiência cardíaca aguda, abordando uma lacuna importante deixada pelo período de pré-inclusão do estudo PARADIGM-HF.^5-7^ No entanto, uma crítica comum em relação a esses estudos diz respeito ao uso de uma dosagem subótima de enalapril (10 mg duas vezes ao dia, em vez do máximo titulável de 20 mg duas vezes ao dia, geralmente recomendado pelas diretrizes de insuficiência cardíaca), em comparação com uma dosagem completa de sacubitril/valsartana (97/103 mg duas vezes ao dia).

Com a finalidade de abordar essa controvérsia e fornecer uma comparação mais equitativa, o presente estudo visou comparar os desfechos clínicos de pacientes com ICFEr tratados com as doses máximas recomendadas pelas diretrizes de sacubitril/valsartana (97/103 mg duas vezes ao dia) versus enalapril (20 mg duas vezes ao dia) ou losartana (100 mg ao dia). O desenho do estudo, os grupos de comparação e os desfechos primários são apresentados na Figura Central.

## Métodos

### Desenho do estudo, população e aspectos éticos

O presente estudo analisou dados de uma coorte observacional em andamento de 2.314 pacientes com insuficiência cardíaca que receberam atendimento entre 1 de agosto de 2010 e 30 de setembro de 2024 em seis hospitais públicos terciários em uma grande região metropolitana. Com base no censo demográfico nacional mais recente e em estudos epidemiológicos anteriores, a coorte abrangeu aproximadamente 8,4% dos indivíduos com insuficiência cardíaca na região do estudo.^8,9^

A coorte incluiu pacientes com 18 anos ou mais que atendiam aos critérios de Framingham para insuficiência cardíaca.^10^ De 2 de janeiro de 2021 a 31 de janeiro de 2024, um total de 931 pacientes com insuficiência cardíaca foram incluídos prospectivamente por meio de uma entrevista clínica estruturada durante o seguimento ambulatorial, visitas hospitalares para exames de imagem cardíaca e internações. De 1 de outubro de 2023 a 30 de setembro de 2024, outros 1.383 pacientes com insuficiência cardíaca registrados no centro de referência local foram incluídos retrospectivamente por meio da revisão de prontuários eletrônicos. Os pacientes tiveram seu histórico médico avaliado desde o diagnóstico de insuficiência cardíaca, que foi considerado o período basal dos participantes, com dados de acompanhamento de 1 de agosto de 2010 a 30 de setembro de 2024. Durante a extração e a revisão dos dados, tivemos acesso a informações que poderiam identificar participantes individuais. Os dados foram acessados para fins de pesquisa entre 1 de janeiro de 2021 e 1 de setembro de 2024, de acordo com as aprovações concedidas pelos Comitês de Ética em Pesquisa.

Dos 2.314 pacientes da coorte, o presente estudo analisou apenas aqueles com fração de ejeção reduzida (≤ 40%) (n = 903). Adicionalmente, indivíduos foram excluídos pelos seguintes motivos: dados de prescrição indisponíveis ou incompletos (n = 180); uso de outro tipo de IECA ou BRA (n = 17); e uso de doses submáximas dos medicamentos investigados (n = 452). A coorte final para a análise primária abrangeu 254 pacientes elegíveis, conforme descrito no fluxograma STROBE (Figura 1). Também foi realizada uma análise de sensibilidade, incluindo os 452 pacientes que receberam doses submáximas dos medicamentos investigados.

**Figura 1:**
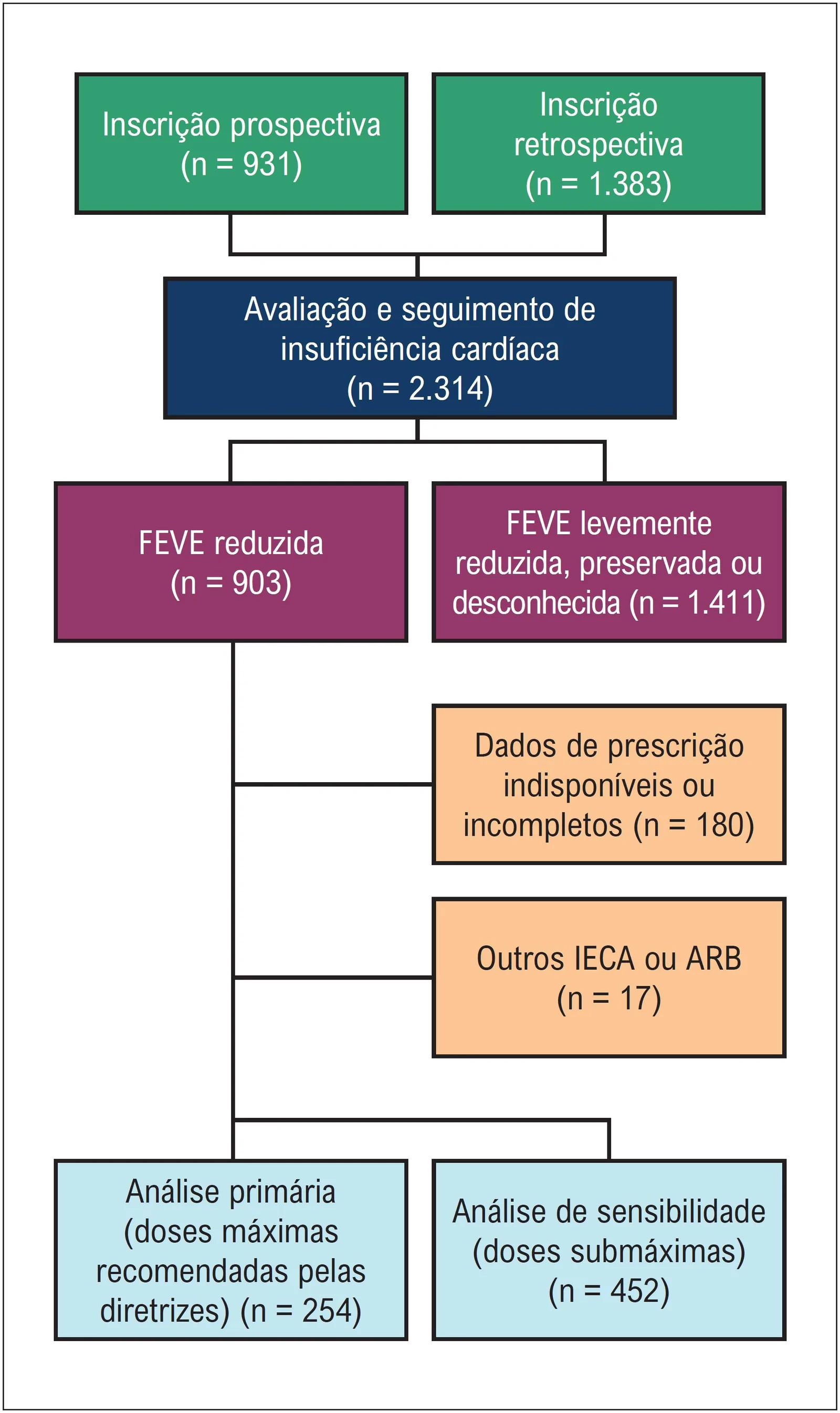
Fluxograma STROBE. BRA: bloqueador do receptor de angiotensina II; FE: fração de ejeção; IECA: inibidor da enzima conversora de angiotensina.

A metodologia do estudo foi aprovada pelos Comitês de Ética em Pesquisa das instituições participantes (números de aprovação: 35543020.1.0000.8153 e 72838823.5.0000.5553). O consentimento livre e esclarecido foi obtido de todos os participantes por meios físicos ou digitais, exceto para os participantes incluídos retrospectivamente que faleceram durante o acompanhamento ou foram considerados incontactáveis após duas tentativas de contato telefônico em dias diferentes, para os quais foi concedida uma dispensa pelos Comitês de Ética em Pesquisa.

### Coleta e definições dos dados

Foi utilizada a plataforma REDCap para a coleta de dados. As seguintes categorias de dados foram coletadas: dados demográficos, comorbidades, farmacoterapia, parâmetros laboratoriais e desfechos clínicos. O desfecho primário foi a combinação de hospitalização por todas as causas ou mortalidade. Os desfechos secundários incluíram eventos cardiovasculares adversos maiores de três pontos (3p-MACE; definidos como a combinação de infarto do miocárdio não fatal, acidente vascular cerebral não fatal ou morte cardiovascular) e progressão da classe funcional da New York Heart Association (NYHA) (sem alteração, melhora ou piora).

### Análise estatística

As análises estatísticas foram realizadas usando o RStudio versão 4.4.2 para Windows. As variáveis categóricas foram expressas como contagens e porcentagens e comparadas usando o teste qui-quadrado de Pearson com correção de continuidade (correção de Yates) ou o teste exato de Fisher. Dado o número relativamente pequeno de eventos em alguns subgrupos, um limiar pragmático baseado nas frequências observadas (≥ 10 vs. < 10 ocorrências) foi adotado para orientar a seleção do teste, com a correção de Yates aplicada para mitigar o risco de superestimar a significância estatística em amostras pequenas. A normalidade das variáveis contínuas com < 50 casos foi avaliada pelo teste de Shapiro–Wilk, e a das variáveis com ≥ 50 casos foi avaliada por inspeção gráfica (histograma, gráfico Q–Q e diagrama de caixa). As variáveis contínuas com distribuição normal foram expressas como média ± desvio padrão e comparadas usando o teste t de Student independente com ajuste de variância. As variáveis contínuas com distribuição não normal foram expressas como mediana e intervalo interquartil (IIQ) e comparadas usando o teste de Mann–Whitney.

Para levar em consideração as diferenças basais entre os grupos de tratamento, inerentes aos estudos observacionais, realizamos o pareamento por escore de propensão (PSM). Para avaliar o impacto do sacubitril/valsartana em desfechos específicos, estimamos o efeito médio do tratamento nos tratados (ATET). Para minimizar o viés devido a covariáveis ausentes, foi realizada a imputação múltipla antes da estimativa do escore de propensão.

A imputação multivariada foi conduzida utilizando o pacote mice. Variáveis contínuas e binárias com até 20% de dados ausentes foram imputadas, como um limiar pragmático e baseado em dados para equilibrar a redução do viés e a estabilidade da estimativa, exceto para: sexo, idade, desfechos e os critérios de inclusão (fração de ejeção do ventrículo esquerdo [FEVE], uso de sacubitril/valsartana, uso de enalapril e uso de losartana). A regressão logística foi usada para a imputação de variáveis binárias e a imputação por média preditiva (*predictive mean matching*) para variáveis contínuas. Variáveis categóricas com mais de 2 categorias não foram imputadas devido à distribuição extrema na maioria delas (por exemplo, muito poucos participantes asiáticos e indígenas para a variável raça). Esse processo reduziu a já baixa porcentagem de células ausentes nas variáveis imputadas de 3,53% para 0,42%. Os escores de propensão foram estimados após a imputação, e o pareamento foi realizado dentro dos conjuntos de dados imputados. As regras de Rubin foram aplicadas para agrupar os valores de p entre as imputações nas análises subsequentes. Foram considerados significativos valores de p < 0,05.

Dado o grande número de variáveis em nosso conjunto de dados, a regressão LASSO (operador de seleção e encolhimento absoluto mínimo) foi empregada para orientar a seleção de covariáveis para o PSM. O pacote MatchIt foi usado para realizar o PSM por meio do método genético, escolhido por sua robustez e eficiência. O ATET foi estimado por regressão logística ponderada e foi restrito a eventos com mais de 10 ocorrências.

## Resultados

Entre agosto de 2010 e setembro de 2024, 254 pacientes com ICFEr foram atendidos em seis hospitais públicos na região do estudo. Um total de 115 pacientes (45,3%) recebeu prescrição de sacubitril/valsartana 97/103 mg duas vezes ao dia, 33 (13,0%) de enalapril 20 mg duas vezes ao dia e 106 (41,7%) de losartana 100 mg ao dia. O tempo mediano de seguimento da coorte do estudo foi de 23,9 meses (IIQ 11,9–41,1).

### Características da população

A população foi composta por 61,4% de pacientes do sexo masculino, com idade média de 63,6 ± 12,3 anos. Quando estratificada por tratamento, não foram observadas diferenças significativas em relação a sexo, idade, composição corporal ou raça (Tabela 1). As comorbidades também foram, em geral, semelhantes entre os grupos. Os indivíduos em uso de sacubitril/valsartana apresentaram FEVE menor (Tabela 1).

**Tabela 1:** Características da população para sacubitril/valsartana em comparação com enalapril ou losartana

Variável			Análises principais			Análises de sensibilidade		
			Enalapril 20 mg duas vezes ao dia ou losartana 100 mg ao dia (n = 139)	Sacubitril/valsartana 97/103 mg duas vezes ao dia (n = 115)	Valor p	Dose submáxima de enalapril ou losartana (n = 297)	Dose submáxima de sacubitril/valsartana (n = 155)	Valor p
**Sexo masculino, n (%)**			85 (61,2%)	71 (61,7%)	1,000*	183 (61,6%)	95 (61,3%)	1,000*
**Idade (anos), M ± DP**			64,6 ± 13,2	62,3 ± 11,2	0,130‡	61,3 ± 13,3	61,6 ± 13,3	0,816‡
	< 65 anos, n (%)		65 (46,8%)	68 (59,1%)	0,066*	186 (62,6%)	85 (54,8%)	0,133*
	65–85 anos, n (%)		68 (48,9%)	46 (40,0%)	0,195*	101 (34%)	65 (41,9%)	0,119*
	≥ 85 anos, n (%)		6 (4,3%)	1 (0,9%)	0,132†	10 (3,4%)	5 (3,2%)	1,000†
**IMC (kg/m^2^), M ± DP**			28,62 ± 6,21	28,82 ± 5,68	0,827‡	26,36 ± 5,64	26,95 ± 5,34	0,382‡
**Raça**								
	Multirracial, n (%)		24 (30,4%)	17 (25,4%)	0,627*	24 (19,5%)	28 (30,1%)	0,100*
	Branca, n (%)		10 (12,7%)	5 (7,5%)	0,414†	15 (12,2%)	5 (5,4%)	0,101†
	Preta, n (%)		44 (55,7%)	45 (67,2%)	0,213*	82 (66,7%)	59 (63,4%)	0,727*
	Outras, n (%)		1 (1,3%)	0 (0,0%)	1,000†	2 (1,6%)	1 (1,1%)	1,000†
**Comorbidades**								
	IMC ≥ 30 kg/m^2^, n (%)		44 (31,9%)	39 (33,9%)	0,835*	66 (22,3%)	34 (21,9%)	1,000*
	Hipertensão, n (%)		108 (77,7%)	78 (67,8%)	0,104*	167 (56,2%)	92 (59,4%)	0,591*
	Diabetes mellitus, n (%)		67 (48,2%)	54 (47,0%)	0,943*	108 (36,4%)	50 (32,3%)	0,444*
	Dislipidemia, n (%)		79 (56,8%)	70 (60,9%)	0,602*	137 (46,4%)	90 (58,4%)	0,021*
	Histórico de tabagismo, n (%)		58 (41,7%)	37 (32,2%)	0,151*	111 (37,4%)	59 (38,1%)	0,967*
	Hipotireoidismo, n (%)		13 (9,4%)	20 (17,4%)	0,087*	40 (17,3%)	20 (12,9%)	0,303*
	Fibrilação atrial, n (%)		18 (13,2%)	14 (12,2%)	0,951*	60 (20,5%)	34 (22,2%)	0,759*
	Apneia obstrutiva do sono, n (%)		3 (2,2%)	3 (2,6%)	1,000†	3 (1,3%)	3 (1,9%)	0,688†
	Asma, n (%)		7 (5,0%)	5 (4,3%)	1,000†	10 (4,3%)	9 (5,8%)	0,632†
	DPOC, n (%)		12 (8,6%)	6 (5,2%)	0,334†	19 (6,4%)	8 (5,2%)	0,680†
	Uso de álcool, n (%)		27 (19,9%)	30 (26,1%)	0,306*	70 (23,9%)	42 (27,5%)	0,479*
	Histórico de câncer, n (%)		8 (5,8%)	3 (2,6%)	0,354†	18 (7,8%)	5 (3,2%)	0,079†
**Lesão de órgão-alvo**								
	FEVE (%), Mdn (IIQ)		33,0 (27,0–37,0)	29,0 (24,0–35,0)	<0,001§	31,0 (24,0–36,0)	27,0 (22,0–31,0)	<0,001§
	Etiologia da insuficiência cardíaca							
		Isquêmica, n (%)	55 (39,6%)	51 (44,3%)	0,521*	83 (27,9%)	55 (35,7%)	0,112*
		Hipertensiva, n (%)	47 (33,8%)	34 (29,6%)	0,557*	61 (26,4%)	39 (25,3%)	0,906*
		Cardiomiopatia chagásica, n (%)	26 (18,7%)	18 (15,7%)	0,636*	63 (27,3%)	46 (29,9%)	0,661*
		Cardiomiopatia dilatada, n (%)	25 (18,0%)	24 (20,9%)	0,674*	42 (18,2%)	24 (15,6%)	0,600*
		Valvar, n (%)	5 (3,6%)	4 (3,5%)	1,000†	16 (6,9%)	11 (7,1%)	1,000*
	Doença renal crônica, n (%)		49 (35,3%)	29 (25,2%)	0,112*	78 (26,4%)	44 (28,6%)	0,711*
		Estágio terminal, n (%)	1 (0,7%)	1 (0,9%)	1,000†	5 (1,7%)	1 (0,6%)	0,669†
	Doença arterial coronariana, n (%)		51 (36,7%)	48 (41,7%)	0,489*	93 (31,3%)	49 (31,6%)	1,000*
		IM prévio, n (%)	43 (31,6%)	45 (39,1%)	0,267*	77 (26,2%)	41 (26,8%)	0,980*
	AVC prévio, n (%)		19 (13,7%)	11 (9,6%)	0,416*	46 (15,5%)	19 (12,3%)	0,431*
**Farmacoterapia**								
	Betabloqueadores, n (%)		130 (93,5%)	108 (93,9%)	1,000*	282 (94,9%)	146 (94,2%)	0,905*
	Inibidores de SGLT2, n (%)		46 (33,1%)	83 (72,2%)	<0,001*	94 (40,7%)	111 (71,6%)	<0,001*
	ARM, n (%)		108 (77,7%)	104 (90,4%)	0,011*	241 (81,1%)	143 (92,3%)	0,003*
	Furosemida, n (%)		81 (58,3%)	62 (53,9%)	0,568*	127 (55,0%)	95 (61,3%)	0,261*
	Tiazídicos, n (%)		17 (12,2%)	7 (6,1%)	0,131	13 (5,6%)	7 (4,5%)	0,815†
	BCC, n (%)		22 (15,8%)	1 (0,9%)	<0,001†	13 (4,4%)	4 (2,6%)	0,440†
	Hidralazina, n (%)		12 (8,6%)	5 (4,3%)	0,212†	6 (2,6%)	6 (3,9%)	0,555†
	Nitratos, n (%)		17 (12,2%)	6 (5,2%)	0,077†	11 (3,7%)	6 (3,9%)	1,000†
	Amiodarona, n (%)		15 (10,8%)	24 (20,9%)	0,041*	52 (17,5%)	45 (29,0%)	0,007*
	Ivabradina, n (%)		6 (4,4%)	13 (11,3%)	0,054†	4 (1,4%)	12 (7,8%)	0,001†
	Digoxina, n (%)		8 (5,8%)	7 (6,1%)	1,000†	32 (10,8%)	19 (12,3%)	0,752*
	Aspirina, n (%)		72 (51,8%)	46 (40,0%)	0,080*	93 (31,4%)	47 (30,3%)	0,895*
	Antagonistas de P2Y12, n (%)		19 (13,7%)	13 (11,3%)	0,707*	24 (8,1%)	7 (4,5%)	0,174†
	Anticoagulantes, n (%)		28 (20,1%)	35 (30,4%)	0,081*	105 (35,5%)	52 (33,8%)	0,798*
	Estatinas, n (%)		101 (72,7%)	85 (73,9%)	0,935*	193 (65,0%)	94 (60,6%)	0,420*
	Ezetimiba, n (%)		7 (5,1%)	10 (8,7%)	0,318†	13 (5,7%)	11 (7,2%)	0,719*
	Fibratos, n (%)		1 (0,7%)	4 (3,5%)	0,182†	5 (2,2%)	1 (0,7%)	0,408†
	Metformina, n (%)		47 (33,8%)	38 (33,0%)	1,000*	49 (16,5%)	21 (13,5%)	0,493*
	Sulfonilureias, n (%)		15 (10,8%)	8 (7,0%)	0,381†	14 (6,1%)	3 (1,9%)	0,074†
	Insulina, n (%)		25 (18,0%)	15 (13,0%)	0,366*	35 (11,8%)	14 (9,0%)	0,456*
**Parâmetros laboratoriais**								
	Creatinina (mg/dL), Mdn (IIQ)		1,19 (0,98–1,51)	1,19 (0,98–1,51)	0,948§	1,13 (0,90–1,39)	1,12 (0,92–1,46)	0,447§
	TFG (mL/min), M ± DP		68,3 ± 20,4	71,8 ± 21,0	0,178‡	74,1 ± 22,7	73,1 ± 21,7	0,675‡
	Sódio (mEq/L), M ± DP		139,2 ± 5,2	140,4 ± 3,3	0,037‡	138,4 ± 4,3	139,6 ± 3,6	0,002‡
	Potássio (mEq/L), M ± DP		4,62 ± 0,54	4,64 ± 0,48	0,847‡	4,61 ± 0,60	4,55 ± 0,54	0,232‡
	Glicemia (mg/dL), Mdn (IIQ)		101,0 (89,0–118,0)	101,0 (89,5–129,5)	0,629§	98,5 (85,0–123,7)	95,0 (85,0–114,5)	0,256§
	Hemoglobina glicada (%), Mdn (IIQ)		6,10 (5,62–7,15)	6,10 (5,60–7,20)	0,925§	6,0 (5,6–6,5)	5,90 (5,6–6,4)	0,374§
	Colesterol total (mg/dL), M ± DP		166,7 ± 56,6	164,8 ± 51,8	0,781‡	168,5 ± 47,1	171,7 ± 50,0	0,509‡
	Colesterol HDL (mg/dL), M ± DP		96,0 ± 53,8	100,2 ± 39,4	0,480‡	104,3 ± 49,2	102,3 ± 42,2	0,650‡
	Colesterol LDL (mg/dL), M ± DP		43,9 ± 15,1	41,3 ± 12,7	0,141‡	45,2 ± 13,2	45,1 ± 15,2	0,985‡
	Triglicerídeos (mg/dL), Mdn (IIQ)		133,0 (89,0–179,0)	129,0 (87,0–182,0)	0,993§	116,0 (78,0–175,0)	119,0 (81,0–165,0)	0,897§
	BNP (pg/mL), Mdn (IIQ)		426,0 (258,0–680,0)	334,0 (143,5–1,771,5)	1,000§	242,7 (68,5–953,6)	199,0 (105,0–1,670,5)	0,579§
	AST (U/L), Mdn (IIQ)		22,0 (18,0–29,0)	23,0 (17,0–29,0)	0,915§	25,0 (19,0–34,0)	24,0 (19,0–30,0)	0,337§
	ALT (U/L), Mdn (IIQ)		21,0 (16,0–29,7)	22,0 (16,0–32,5)	0,706§	22,0 (15,0–34,0)	22,0 (16,0–31,0)	0,942§

* Teste qui-quadrado de Pearson com correção de continuidade

† Teste exato de Fisher

‡ teste t de Student independente com ajuste de variância

§ teste de Mann–Whitney.

ALT: alanina aminotransferase; ARM: antagonistas dos receptores de mineralocorticoides; AST: aspartato aminotransferase; AVC: acidente vascular cerebral; BCC: bloqueadores dos canais de cálcio; BMI: índice de massa corporal; BNP: peptídeo natriurético tipo B; DP: desvio padrão; DPOC: doença pulmonar obstrutiva crônica; GFR: taxa de filtração glomerular; HDL: lipoproteína de alta densidade; IIQ: intervalo interquartil; IM: infarto do miocárdio; LDL: lipoproteína de baixa densidade; FEVE: fração de ejeção do ventrículo esquerdo; M: média; Mdn: mediana; SGLT2: cotransportador de sódio-glicose 2.

Em relação à farmacoterapia para insuficiência cardíaca, o grupo sacubitril/valsartana recebeu com mais frequência inibidores do cotransportador de sódio-glicose 2 (SGLT2) e antagonistas dos receptores de mineralocorticoides (ARM). Esses indivíduos também apresentaram menor probabilidade de receber bloqueadores dos canais de cálcio (BCC) e maior probabilidade de receber amiodarona (Tabela 1). Não foram observadas diferenças significativas para outros medicamentos (betabloqueadores, diuréticos, vasodilatadores, outros antiarrítmicos, antidiabéticos, hipolipemiantes e antitrombóticos). A única diferença estatisticamente significativa em relação aos parâmetros laboratoriais foi um nível sérico ligeiramente mais elevado de sódio no grupo sacubitril/valsartana (Tabela 1).

### Pareamento por escore de propensão

Com base no conhecimento clínico e nas diferenças estatisticamente significativas na análise descritiva, as seguintes covariáveis candidatas foram inseridas na regressão LASSO: sexo, idade, índice de massa corporal (IMC), FEVE, taxa de filtração glomerular (TFG), fatores de risco cardiovascular clássicos (obesidade, hipertensão, diabetes mellitus tipo 2, dislipidemia, histórico de tabagismo), as principais etiologias da insuficiência cardíaca (isquêmica, hipertensiva, cardiomiopatia chagásica, cardiomiopatia dilatada, valvar), outros medicamentos para insuficiência cardíaca (betabloqueadores, inibidores de SGLT2 e ARM), antiarrítmicos, diuréticos, antidiabéticos, hipolipemiantes, antitrombóticos e parâmetros laboratoriais metabólicos (hemoglobina glicada, perfil lipídico). A variável de tratamento foi definida como o uso de sacubitril/valsartana e a variável de desfecho como a combinação de hospitalização ou mortalidade.

A validação cruzada LASSO retornou um lambda mínimo de 0,052, com os seguintes coeficientes: FEVE, histórico de tabagismo, uso de inibidor de SGLT2, uso de BCC e uso de amiodarona. Os participantes foram pareados com base em seu escore de propensão para o uso de sacubitril/valsartana. As seguintes covariáveis foram utilizadas para o cálculo do escore de propensão: idade, FEVE, histórico de tabagismo, uso de inibidor de SGLT2, uso de ARM, uso de BCC e uso de amiodarona. A diferença padronizada média para a distância foi de 1,27 antes do pareamento e de 0,07 após o pareamento (Tabela Suplementar 1). A diferença padronizada média para as covariáveis variou de −1,61 a 0,87 antes do pareamento e de −0,07 a 0,07 após o pareamento. Antes do pareamento, a população era composta por 115 indivíduos tratados com sacubitril/valsartana 97/103 mg duas vezes ao dia e 139 controles (tratados com enalapril 20 mg duas vezes ao dia ou losartana 100 mg ao dia) sem dados faltantes nas covariáveis. O processo de pareamento resultou na perda de 60 controles, mas aumentou significativamente a comparabilidade entre os grupos.

### Desfechos

Durante o seguimento mediano de 23,9 meses (IIQ 11,9–41,1), ocorreram 70 eventos do desfecho primário (56 primeiras hospitalizações e 32 óbitos). Houve uma redução nominal, mas não estatisticamente significativa, na combinação de hospitalização ou mortalidade no grupo sacubitril/valsartana (Tabela 2). Resultados semelhantes foram observados para hospitalização, mortalidade por todas as causas e 3p-MACE (Tabela 2). A exceção notável foi a progressão da classe funcional da NYHA, na qual a melhora foi mais frequente no grupo sacubitril/valsartana (Tabela 2).

**Tabela 2:** Desfechos após pareamento por escore de propensão para sacubitril/valsartana em comparação com enalapril ou losartana

Variável			Análises principais			Análises de sensibilidade		
			Enalapril 20 mg duas vezes ao dia ou losartana 100 mg ao dia (n = 79)	Sacubitril/valsartana 97/103 mg duas vezes ao dia (n = 115)	Valor p	Dose submáxima de enalapril ou losartana (n=132)	Dose submáxima de sacubitril/valsartana (n=155)	Valor p
**Hospitalização ou mortalidade, n (%)**			31 (39,7%)	39 (33,9%)	0,500*	45 (34,9%)	55 (35,9%)	0,951*
	Hospitalização ou mortalidade por insuficiência cardíaca, n (%)		12 (16,0%)	19 (16,5%)	1,000*	24 (18,5%)	26 (17,1%)	0,888*
	≥ 1 hospitalização, n (%)		24 (30,8%)	32 (27,8%)	0,779*	38 (29,2%)	41 (26,8%)	0,748*
	≥ 2 hospitalizações, n (%)		5 (6,4%)	6 (5,2%)	0,759†	18 (13,8%)	12 (7,8%)	0,150*
	≥ 3 hospitalizações, n (%)		2 (2,6%)	3 (2,6%)	1,000†	6 (4,6%)	8 (5,2%)	1,000†
	≥ 4 hospitalizações, n (%)		1 (1,3%)	3 (2,6%)	0,649†	1 (0,8%)	3 (2,0%)	0,627†
	Hospitalização por insuficiência cardíaca, n (%)		9 (12,0%)	14 (12,2%)	1,000†	19 (14,6%)	18 (11,8%)	0,610*
**Mortalidade por todas as causas, n (%)**			15 (19,2%)	17 (14,8%)	0,536*	31 (24%)	29 (19,0%)	0,373*
	Mortalidade CV, n (%)		9 (12,2%)	15 (13,2%)	1,000†	23 (18%)	21 (14,2%)	0,490*
		Mortalidade por IM, n (%)	2 (2,7%)	2 (1,8%)	0,647†	1 (0,8%)	1 (0,7%)	1,000†
		Mortalidade por AVC, n (%)	0 (0,0%)	1 (0,9%)	1,000†	1 (0,8%)	3 (2,0%)	0,626†
		Mortalidade por insuficiência cardíaca, n (%)	6 (8,1%)	9 (7,9%)	1,000†	18 (14,1%)	15 (10,1%)	0,414*
		Mortalidade por outra causa CV, n (%)	1 (1,4%)	3 (2,6%)	1,000†	3 (2,3%)	2 (1,4%)	0,666†
**Mortalidade não CV, n (%)**			4 (5,4%)	3 (2,6%)	0,436†	9 (7,0%)	4 (2,7%)	0,152†
	Mortalidade por causa infecciosa, n (%)		2 (2,7%)	2 (1,8%)	0,647†	5 (3,9%)	2 (1,4%)	0,256†
		Mortalidade por COVID-19, n (%)	1 (1,4%)	0 (0,0%)	0,394†	3 (2,3%)	1 (0,7%)	0,340†
**3p-MACE, n (%)**			12 (16,2%)	17 (14,9%)	0,972*	28 (21,9%)	23 (15,5%)	0,231*
	IM não fatal, n (%)		2 (2,6%)	1 (0,9%)	0,567†	3 (2,3%)	3 (2,0%)	1,000†
	AVC não fatal, n (%)		2 (2,6%)	1 (0,9%)	0,567†	6 (4,6%)	4 (2,6%)	0,521†
Progressão da classe funcional NYHA								
	Sem alteração, n (%)		44 (64,7%)	52 (48,1%)	0,046*	65 (55,1%)	88 (60,3%)	0,469*
	Melhora, n (%)		15 (22,1%)	46 (42,6%)	0,009*	34 (28,8%)	43 (29,5%)	1,000*
	Piora, n (%)		9 (13,2%)	10 (9,3%)	0,459†	19 (16,1%)	15 (10,3%)	0,222*

* Teste qui-quadrado de Pearson com correção de continuidade

† Teste exato de Fisher.

AVC: acidente vascular cerebral; CV: cardiovascular; MACE: evento cardiovascular adverso maior; IM: infarto do miocárdio; NYHA: New York Heart Association.

A análise do ATET confirmou esses achados, não demonstrando reduções significativas para o desfecho primário ou seus componentes (Tabela 3). Também revelou uma associação ainda mais forte para a melhora da classe funcional da NYHA (Tabela 3).

**Tabela 3:** Efeito médio do tratamento nos tratados com sacubitril/valsartana em comparação com enalapril ou losartana (doses máximas)

Variável		Análises principais				Análises de sensibilidade			
		β	SE	Valor p	OR (IC 95%)	β	SE	Valor p	OR (IC 95%)
**Hospitalização ou mortalidade**		−0,431	0,311	0,165*	0,650 (0,354–1,195)	0,202	0,271	0,455*	1,224 (0,702–2,081)
	Hospitalização ou mortalidade por insuficiência cardíaca	−0,236	0,391	0,546*	0,790 (0,367–1,700)	0,025	0,336	0,941*	1,025 (0,531–1,980)
	≥ 1 hospitalização	−0,286	0,324	0,377*	0,751 (0,398–1,418)	0,001	0,290	0,998*	1,001 (0,567–1,765)
	≥ 2 hospitalizações	−0,254	0,628	0,686*	0,776 (0,227–2,657)	−0,667	0,399	0,094*	0,513 (0,235–1,121)
	Hospitalização por insuficiência cardíaca	−0,428	0,421	0,310*	0,652 (0,286–1,488)	−0,230	0,367	0,531*	0,795 (0,387–1,631)
**Mortalidade por todas as causas**		−0,214	0,410	0,602*	0,808 (0,362–1,802)	−0,049	0,321	0,879*	0,952 (0,508–1,786)
	Mortalidade CV	0,057	0,465	0,902*	1,059 (0,426–2,635)	−0,127	0,351	0,716*	0,880 (0,443–1,751)
	Mortalidade por insuficiência cardíaca	0,523	0,660	0,428*	1,688 (0,463–6,154)	−0,226	0,396	0,568*	0,797 (0,367–1,733)
**3p-MACE**		−0,193	0,415	0,642*	0,824 (0,365–1,861)	−0,268	0,328	0,415*	0,765 (0,402–1,456)
**Progressão da classe funcional NYHA**									
	Sem alteração	−0,942	0,338	0,005*	0,390 (0,201–0,757)	0,153	0,266	0,564*	1,165 (0,692–1,962)
	Melhora	1,362	0,411	0,001*	3,902 (1,745–8,726)	0,212	0,300	0,480*	1,236 (0,687–2,223)
	Piora	−0,427	0,491	0,385*	0,653 (0,249–1,710)	−0,660	0,367	0,072*	0,517 (0,252–1,061)

* Regressão logística ponderada e log odds (beta).

CV: cardiovascular; IC: intervalo de confiança; NYHA: New York Heart Association; OR: odds ratio; SE: erro padrão; 3p-MACE: eventos cardiovasculares adversos maiores de três pontos.

### Análise de sensibilidade

O presente estudo também avaliou os dados dos 452 pacientes que receberam quaisquer doses submáximas dos medicamentos investigados. Essa população foi 61,5% masculina, com idade média de 61,4 anos e mediana de seguimento de 19,3 (IIQ 8,7–33,7) meses. Um total de 155 pacientes (34,2%) utilizou sacubitril/valsartana, 201 (44,4%) enalapril e 96 (21,2%) losartana. As diferenças nas características da população estratificadas por medicamento foram semelhantes às observadas com doses otimizadas, com FEVE menor e uso mais frequente de inibidores de SGLT2, ARM e amiodarona no grupo sacubitril/valsartana. Após o PSM (Tabela Suplementar 2), não foram observadas diferenças nos desfechos primários ou secundários, o que foi confirmado pela análise do ATET.

## Discussão

O presente estudo utilizou dados de mundo real de uma coorte observacional para comparar os desfechos clínicos de pacientes com ICFEr que receberam as doses máximas recomendadas pelas diretrizes de sacubitril/valsartana (97/103 mg duas vezes ao dia) versus enalapril (20 mg duas vezes ao dia) ou losartana (100 mg ao dia). Não foram observadas reduções significativas no desfecho primário, hospitalização ou morte combinadas (*odds ratio* [OR] 0,650, IC 95% 0,354–1,195, p = 0,165), nem em seus componentes, primeira hospitalização (OR 0,751, IC 95% 0,398–1,418, p = 0,377) e mortalidade por todas as causas (OR 0,808, IC 95% 0,362–1,802, p = 0,602). Esses achados não corroboram a redução de hospitalizações ou mortalidade com o uso de sacubitril/valsartana em comparação com enalapril ou losartana nas doses máximas recomendadas pelas diretrizes.

Após o ensaio PARADIGM-HF, estudos subsequentes comparando sacubitril/valsartana com doses equivalentes de IECA ou BRA não encontraram diferenças estatisticamente significativas entre os grupos em pacientes com FEVE preservada ou reduzida.^11-13^ No ensaio LIFE, entre pacientes com ICFEr avançada, sacubitril/valsartana não demonstrou redução adicional de NT-proBNP em comparação com a valsartana (área sob a curva 0,95, IC 95% 0,84–1,08, p = 0,45).^11^ Da mesma forma, o estudo PARADISE-MI, que incluiu pacientes com disfunção ventricular esquerda após infarto do miocárdio, não demonstrou superioridade de sacubitril/valsartana sobre ramipril na prevenção de morte cardiovascular ou eventos de insuficiência cardíaca (*hazard ratio* 0,90, IC 95% 0,78–1,04, p = 0,17).^12^ Além disso, uma metanálise recente comparou sacubitril/valsartana com IECA e BRA em doses equivalentes e não encontrou reduções significativas na mortalidade por todas as causas (*risk ratio* 0,90, IC 95% 0,76–1,07, p = 0,38, I² = 0%).^13^ Esses estudos também apoiam a hipótese de que os resultados favoráveis observados com sacubitril/valsartana no estudo PARADIGM-HF podem ter sido influenciados, pelo menos parcialmente, pelo uso de um regime de controle subótimo.

Sacubitril/valsartana foi fortemente associado à melhora da classe funcional da NYHA (OR 3,902, IC 95% 1,745–8,726, p = 0,001), mesmo na ausência de reduções estatisticamente significativas na hospitalização ou mortalidade. Esse achado pode estar relacionado ao aumento dos efeitos natriuréticos e hemodinâmicos da inibição da neprilisina. É importante ressaltar que não foram observadas diferenças significativas no uso de diuréticos entre os grupos e que o PSM alcançou um equilíbrio quase perfeito em relação aos inibidores de SGLT2 (72,1% vs. 70,0%) e ARM (90,4% vs. 91,1%). Embora o equilíbrio das covariáveis tenha melhorado após o pareamento, não pode ser totalmente excluída a presença de fatores de confusão residuais. Essa hipótese foi posteriormente avaliada por meio de regressão logística multivariada (considerando idade, sexo, FEVE, TFG, medicamentos para insuficiência cardíaca e diuréticos), que mostrou resultados semelhantes (sacubitril/valsartana com OR de 2,805, IC 95% 1,275–6,172, p = 0,010) (Tabela Suplementar 3). Isso está de acordo com outros estudos que mostram melhora da classe NYHA e nos escores de qualidade de vida, apesar de não haver reduções significativas na hospitalização ou mortalidade.^4,13^ No entanto, esse achado não foi observado na análise de sensibilidade, possivelmente refletindo maior heterogeneidade na exposição ao tratamento, bem como a natureza subjetiva da classificação da NYHA.

Um último aspecto que merece consideração é o contexto econômico que envolve o uso de sacubitril/valsartana. Considerando as doses máximas recomendadas pelas diretrizes e os preços de varejo encontrados online em farmácias dos Estados Unidos, sacubitril/valsartana custa aproximadamente US$ 600 por mês, enquanto enalapril e losartana estão disponíveis por aproximadamente US$ 25 e US$ 10 por mês, respectivamente. No contexto do estudo, apesar da política de medicamentos genéricos, que permite a produção de medicamentos a baixo custo, sacubitril/valsartana permanece consideravelmente mais caro, custando aproximadamente US$ 55 por mês, em comparação com enalapril e losartana, disponíveis por aproximadamente US$ 2 e US$ 3 por mês, respectivamente. O presente estudo não realizou análise farmacoeconômica ou de custo-efetividade formal. Portanto, essas diferenças de preço devem ser interpretadas apenas como informações contextuais. Mais estudos farmacoeconômicos específicos são necessários para avaliar a custo-efetividade de sacubitril/valsartana em diversos contextos de saúde.

Os achados do presente estudo ressaltam a importância de incorporar evidências de mundo real na avaliação de estratégias terapêuticas. Em cenários cada vez mais complexos, marcados por múltiplas opções farmacológicas, é essencial compreender o desempenho de novas terapias fora do ambiente controlado dos ensaios clínicos. Sacubitril/valsartana deve ser avaliado não apenas quanto à eficácia, mas também em termos de aplicabilidade, custo-benefício e implementação em diferentes contextos de saúde, particularmente em países de baixa e média renda com recursos de saúde limitados. Embora nossos achados forneçam informações relevantes de mundo real, comparações diretas com resultados de ensaios clínicos randomizados devem ser interpretadas com cautela, dada a natureza observacional do presente estudo.

### Limitações

A principal limitação do nosso estudo é o tamanho relativamente pequeno da amostra. A redução nominal no desfecho primário observada no grupo sacubitril/valsartana levanta a questão de se essa diferença atingiria significância estatística em uma coorte maior. Usando o GPower 3.1.9.7, o poder estatístico (1 − erro β) para os desfechos medidos foi excelente (0,893). Isso sugere que o tamanho da nossa amostra foi suficiente para detectar o efeito observado, embora a redução no tamanho efetivo da amostra após o PSM possa ter limitado a detecção de diferenças menores entre os grupos.

O desenho observacional e parcialmente retrospectivo representa outra limitação. Para levar em consideração as diferenças basais entre os grupos de tratamento, realizamos o PSM. Sem esse ajuste, a maior prevalência de inibidores de SGLT2 (72,2% vs. 33,1%, p < 0,001) e de ARM (90,4% vs. 77,7%, p = 0,011) no grupo sacubitril/valsartana poderia ter influenciado substancialmente os desfechos. Embora o equilíbrio das covariáveis tenha melhorado após o pareamento, não pode ser totalmente excluída a presença de fatores de confusão residuais. Ademais, a exclusão de pacientes devido a dados faltantes e o PSM podem ter introduzido viés de seleção e devem ser considerados na interpretação dos achados.

A coleta de dados também apresenta limitações inerentes ao desenho do estudo. A adesão ao tratamento não foi avaliada sistematicamente, particularmente na parte retrospectiva da coorte, o que pode ter influenciado os desfechos observados. Da mesma forma, a determinação dos desfechos foi baseada principalmente em prontuários médicos, com seguimento prospectivo parcial, o que pode ter introduzido variabilidade na coleta de dados.

Algumas considerações metodológicas também devem ser levadas em conta. A combinação de enalapril e losartana em um único grupo comparador pode ter introduzido heterogeneidade farmacológica, embora essa abordagem tenha sido planejada para refletir as estratégias convencionais de bloqueio do sistema renina–angiotensina na prática clínica de rotina. Além disso, os modelos de regressão utilizados avaliaram a ocorrência cumulativa de eventos e não levaram em consideração as diferenças no tempo até o evento, o que pode limitar a avaliação dos padrões temporais dos desfechos.

O longo período de seguimento foi geralmente positivo na medida em que permitiu a ocorrência de mais eventos de desfecho. No entanto, o início do período de observação em agosto de 2010 cria um potencial viés, visto que o grupo sacubitril/valsartana seria mais recente, com melhor acesso a terapias modernas para insuficiência cardíaca (ou seja, inibidores de SGLT2) e outras doenças. Esse problema foi parcialmente mitigado pelo PSM. Para isolar ainda mais o impacto do sacubitril/valsartana, também estimamos o ATET. Embora essas abordagens reduzam os fatores de confusão, o viés temporal residual não pode ser totalmente descartado.

## Conclusão

No presente estudo de mundo real em pacientes com ICFEr, o tratamento com sacubitril/valsartana não foi associado a reduções estatisticamente significativas no desfecho combinado de hospitalização ou morte, quando comparado às doses máximas de enalapril ou losartana recomendadas pelas diretrizes. Da mesma forma, não foram observadas diferenças estatisticamente significativas para os desfechos individuais de hospitalização, mortalidade por todas as causas ou eventos cardiovasculares adversos maiores (3p-MACE).

Apesar de não ter havido impacto nesses eventos clínicos importantes, o tratamento com sacubitril/valsartana foi fortemente associado a uma melhora no estado funcional dos pacientes, conforme avaliado pela classificação da NYHA.

Os achados sugerem que, em um contexto de mundo real, onde as terapias de base são otimizadas, a magnitude do benefício do sacubitril/valsartana na mortalidade e hospitalização pode diferir daquela observada em ensaios clínicos randomizados. Os resultados destacam a necessidade de mais pesquisas para esclarecer o papel de sacubitril/valsartana em subgrupos específicos de pacientes, incluindo estudos dedicados à avaliação de custo-efetividade em diversos contextos de saúde.

## Data Availability

Os conteúdos subjacentes ao texto da pesquisa estão contidos no manuscrito.

## *Material suplementar

Material suplementar Para informação adicional, por favor, clique aqui.
