# Supplementary material for: Sacubitril/Valsartana versus Enalapril ou Losartana em Doses Máximas na Insuficiência Cardíaca com Fração de Ejeção Reduzida: Dados de Mundo Real
Source: Arq Bras Cardiol. 2026 Jul 30;123(7):e20260069. [Article in Portuguese] doi: 10.36660/abc.20260069 (PMC13668448; doi:10.36660/abc.20260069)
Supplement: Supplementary file 1 [file 0066-782x-abc-123-7-e20260069-Suppl01-en.pdf]

Sacubitril/Valsartan Versus Enalapril or Losartan at Maximum Doses in Heart Failure with Reduced Ejection Fraction: Real-World Data

SUPPLEMENTAL DATA

Supplementary table 1. Propensity score matching (PSM) for sacubitril-valsartan compared to either enalapril or losartan (maximum dosages).

Variable	Before matching			After matching		
	Enalapril 20mg twice daily, or Losartan 100mg daily (n=139)	Sacubitril-Valsartan 97/103mg twice daily (n=115)	Std. diff.	Enalapril 20mg twice daily, or Losartan 100mg daily (n=79)	Sacubitril-Valsartan 97/103mg twice daily (n=115)	Std. diff.
Distance, M	0.34	0.59	1.27	0.58	0.59	0.07
Age (years), M	64.68	62.35	-0.21	62.41	62.35	-0.01
LVEF (%), M	31.77	28.81	-0.45	29.29	28.81	-0.07
Smoking history, M	0.42	0.32	-0.20	0.30	0.32	0.06
SGLT2 inhibitor, M	0.33	0.72	0.87	0.70	0.72	0.05
CCBs, M	0.16	0.01	-1.61	0.01	0.01	<0.01
MRAs, M	0.78	0.90	0.43	0.91	0.90	-0.01
Amiodarone, M	0.11	0.21	0.25	0.20	0.21	0.02

M: mean. LVEF: left ventricle ejection fraction. SGLT2: sodium-glucose cotransporter 2. CCBs: calcium channel blockers. MRAs: mineralocorticoid receptor antagonists.

Supplementary table 2. Propensity score matching (PSM) for sacubitril-valsartan compared to either enalapril or losartan (sub-maximum doses).

Variable	Before matching			After matching		
	Sub-maximum Enalapril or Losartan (n=231)	Sub-maximum Sacubitril-Valsartan (n=155)	Std. diff.	Sub-maximum Enalapril or Losartan (n=132)	Sub-maximum Sacubitril-Valsartan (n=155)	Std. diff.
Distance, M	0.33	0.51	0.93	0.49	0.51	0.10
Age (years), M	64.47	61.69	-0.21	62.21	61.69	-0.04
LVEF (%), M	30.05	26.49	-0.57	27.17	26.49	-0.11
Smoking history, M	0.40	0.38	-0.04	0.38	0.38	<0.01
SGLT2 inhibitor, M	0.41	0.72	0.69	0.69	0.72	0.06
CCBs, M	0.03	0.03	-0.03	0.02	0.03	0.06
MRAs, M	0.79	0.92	0.49	0.92	0.92	<0.01
Amiodarone, M	0.20	0.29	0.20	0.29	0.29	<0.01

M: mean. LVEF: left ventricle ejection fraction. SGLT2: sodium-glucose cotransporter 2. CCBs: calcium channel blockers. MRAs: mineralocorticoid receptor antagonists.

Supplementary table 3. Multivariate logistic regression for NYHA class improvement (maximum dosages).

Variable	β	SE	p-value	OR (95%CI)
Age (years)	-0.012	0.020	0.552	0.988 (0.949 - 1.028)
Male sex	0.306	0.408	0.454	1.358 (0.610- 3.020)
LVEF (%)	-0.049	0.028	0.075	0.952 (0.902- 1.005)
GFR (mL/min)	0.004	0.012	0.723	1.004 (0.981 - 1.027)
Sacubitril-valsartan*	1.031	0.402	**0.010**	2.805 (1.275 - 6.172)
Beta-blockers	-0.130	0.736	0.860	0.878 (0.208 - 3.718)
SGLT2 inhibitor	-0.644	0.398	0.105	0.525 (0.241 - 1.145)
MRAs	1.397	0.707	**0.048**	4.042 (1.011 - 16.163)
Thiazides	0.882	0.370	**0.017**	2.415 (1.169 - 4.989)
Furosemide	0.289	0.676	0.668	1.336 (0.355 - 5.020)

*All patients not on sacubitril-valsartan were on either enalapril or losartan. LVEF: left ventricular ejection fraction. GFR: glomerular filtration rate. SGLT2: sodium-glucose linked transporter 2. MRAs: mineralocorticoid receptor antagonists.
