# Supplementary material for: Sacubitril/Valsartana versus Enalapril ou Losartana em Doses Máximas na Insuficiência Cardíaca com Fração de Ejeção Reduzida: Dados de Mundo Real
Source: Arq Bras Cardiol. 2026 Jul 30;123(7):e20260069. [Article in Portuguese] doi: 10.36660/abc.20260069 (PMC13668448; doi:10.36660/abc.20260069)
Supplement: Translation

# Translation: Sacubitril/Valsartan versus Enalapril or Losartan at Maximum Doses in Heart Failure with Reduced Ejection Fraction: Real-World Data

**DOI:** 10.36660/abc.20260069i

**Authors:** Renato de Carvalho Barros, Vinícius C. Fiusa, Gabrielle N. Tim, Victoria T. V. Goulart, Caio Henrique M. Nascimento, Gustavo T. de Moura, Adriana J. B. A. Guimarães, Sergio Henrique Rodolpho Ramalho, Armindo Jreige, Filipe A. Moura, Luiz Sérgio Fernandes de Carvalho

**Affiliations:** 1 Brasília DF Brasil Universidade de Brasília - Faculdade de Ciências Médicas, Brasília, DF – Brasil; 2 Brasília DF Brasil Laboratório de Dados para Qualidade do Cuidado e Pesquisa de Desfechos (LaDaQCOR), Brasília, DF – Brasil; 3 Brasília DF Brasil Grupo de Pesquisa em Doenças Cardiovasculares de Brasília – Escola Superior de Ciências da Saúde (ESCS), Brasília, DF – Brasil; 4 Brasília DF Brasil Escola Superior de Ciências da Saúde (ESCS), Brasília, DF – Brasil; 5 São Paulo SP Brasil Instituto Sírio-libanês de Ensino e Pesquisa, São Paulo, SP – Brasil; 6 Brasília DF Brasil Instituto de Cardiologia do Distrito Federal (ICDF), Brasília, DF – Brasil; 7 New Haven EUA Yale School of Medicine - Section of Cardiovascular Medicine, New Haven – EUA; 8 West Haven EUA VA Connecticut Healthcare System, West Haven – EUA; 9 Taguatinga DF Brasil Universidade Católica de Brasília – Gerontologia, Taguatinga, DF – Brasil

**Keywords:** Systolic Heart Failure, Valsartan, Enalapril

## Abstract

**Background:**

Sacubitril/valsartan reduced mortality and hospitalizations in patients with heart failure with reduced ejection fraction (HFrEF) compared to enalapril in pivotal clinical trials. However, real-world data under optimized dosing remain limited.

**Objective:**

To compare the outcomes of HFrEF patients treated with sacubitril/valsartan versus either enalapril or losartan at guideline-recommended maximum dosages.

**Methods:**

We analyzed data from an observational heart failure cohort (n = 2,314) including patients receiving care between August 2010 and September 2024 at six public hospitals in a large metropolitan area. The primary analysis included 254 HFrEF patients receiving the guideline-recommended maximum dosages of the investigated drugs. A sensitivity analysis included 452 patients receiving any submaximal dosages. The primary outcome was combined hospitalization or death. Baseline differences were adjusted using propensity score matching. A p-value < 0.05 was considered significant.

**Results:**

Among 254 HFrEF patients (mean age 63.6 ± 12.3 years, 61.4% male), 115 received sacubitril/valsartan 97/103mg twice daily, 33 enalapril 20 mg twice daily, and 106 losartan 100 mg daily. Over the median follow-up of 23.9 months (interquartile range 11.9–41.1), there were 70 primary endpoint events (56 first hospitalizations, 32 deaths). Compared to equivalent dosages of enalapril or losartan, sacubitril/valsartan was not associated with significant reductions in combined hospitalization or death (odds ratio 0.650, 95% confidence interval 0.354–1.195, p = 0.165), but it was associated with functional status improvement (odds ratio 3.902, 95% confidence interval 1.745–8.726, p = 0.001).

**Conclusion:**

This real-world study did not demonstrate significant reductions in hospitalization or mortality for sacubitril/valsartan compared with enalapril or losartan at guideline-recommended maximum dosages; however, sacubitril/valsartan was associated with improved functional status.

## Introduction

In the past decade, angiotensin receptor–neprilysin inhibitors (ARNIs) have emerged as a cornerstone treatment of heart failure with reduced ejection fraction (HFrEF). The combination of sacubitril with valsartan promotes vasodilation and natriuresis while counteracting the cardiotoxic effects of angiotensin II.^1^

Current heart failure (HF) guidelines recommend ARNIs over angiotensin-converting enzyme (ACE) inhibitors or angiotensin receptor blockers (ARBs) for patients with HFrEF.^2,3^ This recommendation was initially supported by the PARADIGM-HF trial, which demonstrated a significant reduction in mortality and hospitalizations with sacubitril/valsartan compared to enalapril.^4^ Subsequently, a pooled analysis of data from the PIONEER-HF and PARAGLIDE-HF trials provided further evidence supporting the use of ARNIs in acute settings, addressing a key gap left by the run-in period of the PARADIGM-HF trial.^5-7^ However, a common criticism of these studies lies in the use of a suboptimal dosage of enalapril (10 mg twice daily, rather than the titratable maximum of 20 mg twice daily, generally recommended by HF guidelines), compared to a full dosage of sacubitril/valsartan (97/103 mg twice daily).

**Figure:** MACE: major adverse cardiovascular events; NYHA: New York Heart Association.

To address this controversy and provide a more equitable comparison, the present study aimed to compare clinical outcomes of patients with HFrEF treated with the guideline-recommended maximum dosages of sacubitril/valsartan (97/103 mg twice daily) versus either enalapril (20 mg twice daily) or losartan (100 mg daily). The study design, comparison groups, and primary outcomes are summarized in the Central Illustration.

## Methods

### Study design, population, and ethics

This study analyzed data from an ongoing observational cohort of 2,314 patients with HF who received care between August 1, 2010 and September 30, 2024 at six public tertiary-care hospitals in a large metropolitan region. Based on the most recent national demographic census and on prior epidemiological studies, the cohort encompassed approximately 8.4% of individuals with HF in the study region.^8,9^

The cohort enrolled patients aged 18 years and older who met the Framingham criteria for HF.^10^ From January 2, 2021 until January 31, 2024, a total of 931 patients with HF were enrolled prospectively through structured clinical interview during outpatient follow-up, hospital visits for cardiac imaging studies, and hospitalizations. From October 1, 2023 to September 30, 2024, an additional 1,383 patients with HF registered at the local referral center were enrolled retrospectively through electronic health record review. Patients had their medical history assessed since the diagnosis of HF, which was considered the participants’ baseline, with follow-up data from August 1, 2010 to September 30, 2024. During data extraction and review, we had access to information that could identify individual participants. Data were accessed for research purposes between January 1, 2021 and September 1, 2024, in accordance with the approvals granted by the Institutional Review Boards.

From the 2,314 patients in the cohort, the current study only analyzed those with reduced ejection fraction (≤ 40%) (n = 903). Moreover, individuals were excluded due to the following reasons: unavailable or incomplete prescription data (n = 180); use of another type of ACE inhibitor or ARB (n = 17); and use of submaximal dosages of the investigated drugs (n = 452). The final cohort for the primary analysis encompassed 254 eligible patients, as outlined in the STROBE flowchart (Figure 1). A sensitivity analysis, including the 452 patients on any submaximal dosages of the investigated drugs, was also performed.

**Figure 1:** STROBE flowchart. ACEi: angiotensin-converting enzyme inhibitor; ARB: angiotensin II receptor blocker; EF: ejection fraction.

The study methodology was approved by the Institutional Review Boards of the participating institutions (approval numbers: 35543020.1.0000.8153 and 72838823.5.0000.5553). Informed consent was obtained from all participants by physical or digital means, except for retrospectively enrolled participants who either died during follow-up or were deemed unreachable after two telephone contact attempts on different days, for whom a waiver was granted by the Institutional Review Boards.

### Data collection and definitions

The REDCap platform was used for data capture. The following data categories were collected: demographics, comorbidities, pharmacotherapy, laboratory parameters, and clinical outcomes. The primary outcome was combined all-cause hospitalization or death. Secondary outcomes included three-point major adverse cardiovascular events (3p-MACE; defined as combined non-fatal myocardial infarction, non-fatal stroke, or cardiovascular death), and New York Heart Association (NYHA) class progression (no change, improvement, or worsening).

### Statistical analysis

Statistical analyses were conducted using RStudio version 4.4.2 for Windows. Categorical variables were expressed as counts and percentages and compared using continuity-corrected Pearson's chi-squared test (Yates correction) or Fisher's exact test. Given the relatively small number of events in some subgroups, a pragmatic threshold based on observed frequencies (≥ 10 vs. < 10 occurrences) was adopted to guide test selection, with Yates correction applied to mitigate the risk of overestimating statistical significance in small samples. Continuous variables with < 50 cases had their normality evaluated by the Shapiro–Wilk test, and those with ≥ 50 cases by graphical inspection (histogram, Q–Q plot, and box plot). Normal continuous variables were expressed as mean ± standard deviation and compared using variance-adjusted independent Student's t test. Non-normal continuous variables were expressed as median and interquartile range (IQR) and compared using the Mann–Whitney test.

To account for baseline differences between treatment groups, inherent to observational studies, we performed propensity score matching (PSM). To evaluate the impact of sacubitril/valsartan on specific outcomes, we estimated the average treatment effect on the treated (ATET). To minimize bias due to missing covariates, multiple imputation was performed prior to propensity score estimation.

Multivariate imputation was conducted using the mice package. Continuous and binary variables with up to 20% missing data were imputed, as a pragmatic and data-driven threshold to balance bias reduction and estimate stability, except for: sex, age, outcomes, and the inclusion criteria (left ventricular ejection fraction [LVEF], sacubitril/valsartan use, enalapril use, and losartan use). Logistic regression was used for the imputation of binary variables, and predictive mean matching for continuous variables. Categorical variables with more than 2 categories were not imputed due to the extreme distribution in most of them (e.g., very few Asian and Indigenous participants for the race variable). This process reduced the already low percentage of missing cells in imputed variables from 3.53% to 0.42%. Propensity scores were estimated after imputation, and matching was performed within the imputed datasets. Rubin's rules were applied to pool p-values across imputations in subsequent analyses. A p-value <0.05 was considered significant.

Given the large number of variables in our dataset, LASSO regression (least absolute shrinkage and selection operator) was employed to guide covariate selection for PSM. The MatchIt package was used to perform PSM through the genetic method, chosen for its robustness and efficiency. ATET was estimated by weighted logistic regression and was restricted to events with more than 10 occurrences.

## Results

Between August 2010 and September 2024, 254 patients with HFrEF received care across six public hospitals in the study region. A total of 115 patients (45.3%) were prescribed sacubitril/valsartan 97/103 mg twice daily, 33 (13.0%) enalapril 20 mg twice daily, and 106 (41.7%) losartan 100 mg daily. The median follow-up of this cohort was 23.9 months (IQR 11.9–41.1).

### Population characteristics

The population was 61.4% male, with a mean age of 63.6 ± 12.3 years. When stratified by treatment, no significant differences were observed for sex, age, body composition, or race (Table 1). Comorbidities were also largely similar between groups. Individuals on sacubitril/valsartan had a lower LVEF (Table 1).

**Table 1:** Population characteristics for sacubitril/valsartan compared to either enalapril or losartan

Variable			Main analyses			Sensitivity analyses		
			Enalapril 20 mg twice daily or losartan 100 mg daily (n = 139)	Sacubitril/valsartan 97/103 mg twice daily (n = 115)	p-value	Submaximal enalapril or losartan (n = 297)	Submaximal sacubitril/valsartan (n = 155)	p-value
**Male sex, n (%)**			85 (61.2%)	71 (61.7%)	1.000*	183 (61.6%)	95 (61.3%)	1.000*
**Age (years), M ± SD**			64.6 ± 13.2	62.3 ± 11.2	0.130‡	61.3 ± 13.3	61.6 ± 13.3	0.816‡
	< 65 years, n (%)		65 (46.8%)	68 (59.1%)	0.066*	186 (62.6%)	85 (54.8%)	0.133*
	65–85 years, n (%)		68 (48.9%)	46 (40.0%)	0.195*	101 (34%)	65 (41.9%)	0.119*
	≥ 85 years, n (%)		6 (4.3%)	1 (0.9%)	0.132†	10 (3.4%)	5 (3.2%)	1.000†
**BMI (kg/m2), M ± SD**			28.62 ± 6.21	28.82 ± 5.68	0.827‡	26.36 ± 5.64	26.95 ± 5.34	0.382‡
**Race**								
	Mixed, n (%)		24 (30.4%)	17 (25.4%)	0.627*	24 (19.5%)	28 (30.1%)	0.100*
	White, n (%)		10 (12.7%)	5 (7.5%)	0.414†	15 (12.2%)	5 (5.4%)	0.101†
	Black, n (%)		44 (55.7%)	45 (67.2%)	0.213*	82 (66.7%)	59 (63.4%)	0.727*
	Others, n (%)		1 (1.3%)	0 (0.0%)	1.000†	2 (1.6%)	1 (1.1%)	1.000†
**Comorbidities**								
	BMI ≥ 30 kg/m^2^, n (%)		44 (31.9%)	39 (33.9%)	0.835*	66 (22.3%)	34 (21.9%)	1.000*
	Hypertension, n (%)		108 (77.7%)	78 (67.8%)	0.104*	167 (56.2%)	92 (59.4%)	0.591*
	Diabetes mellitus, n (%)		67 (48.2%)	54 (47.0%)	0.943*	108 (36.4%)	50 (32.3%)	0.444*
	Dyslipidemia, n (%)		79 (56.8%)	70 (60.9%)	0.602*	137 (46.4%)	90 (58.4%)	0.021*
	Smoking history, n (%)		58 (41.7%)	37 (32.2%)	0.151*	111 (37.4%)	59 (38.1%)	0.967*
	Hypothyroidism, n (%)		13 (9.4%)	20 (17.4%)	0.087*	40 (17.3%)	20 (12.9%)	0.303*
	Atrial fibrillation, n (%)		18 (13.2%)	14 (12.2%)	0.951*	60 (20.5%)	34 (22.2%)	0.759*
	Obstructive sleep apnea, n (%)		3 (2.2%)	3 (2.6%)	1.000†	3 (1.3%)	3 (1.9%)	0.688†
	Asthma, n (%)		7 (5.0%)	5 (4.3%)	1.000†	10 (4.3%)	9 (5.8%)	0.632†
	COPD, n (%)		12 (8.6%)	6 (5.2%)	0.334†	19 (6.4%)	8 (5.2%)	0.680†
	Alcohol use, n (%)		27 (19.9%)	30 (26.1%)	0.306*	70 (23.9%)	42 (27.5%)	0.479*
	Cancer history, n (%)		8 (5.8%)	3 (2.6%)	0.354†	18 (7.8%)	5 (3.2%)	0.079†
**End-organ damage**								
	LVEF (%), Mdn (IQR)		33.0 (27.0–37.0)	29.0 (24.0–35.0)	<0.001§	31.0 (24.0–36.0)	27.0 (22.0–31.0)	<0.001§
	Heart failure etiology							
		Ischemic, n (%)	55 (39.6%)	51 (44.3%)	0.521*	83 (27.9%)	55 (35.7%)	0.112*
		Hypertensive, n (%)	47 (33.8%)	34 (29.6%)	0.557*	61 (26.4%)	39 (25.3%)	0.906*
		Chagas cardiomyopathy, n (%)	26 (18.7%)	18 (15.7%)	0.636*	63 (27.3%)	46 (29.9%)	0.661*
		Dilated cardiomyopathy, n (%)	25 (18.0%)	24 (20.9%)	0.674*	42 (18.2%)	24 (15.6%)	0.600*
		Valvular, n (%)	5 (3.6%)	4 (3.5%)	1.000†	16 (6.9%)	11 (7.1%)	1.000*
	Chronic kidney disease, n (%)		49 (35.3%)	29 (25.2%)	0.112*	78 (26.4%)	44 (28.6%)	0.711*
		End-stage, n (%)	1 (0.7%)	1 (0.9%)	1.000†	5 (1.7%)	1 (0.6%)	0.669†
	Coronary arterial disease, n (%)		51 (36.7%)	48 (41.7%)	0.489*	93 (31.3%)	49 (31.6%)	1.000*
		Prior MI, n (%)	43 (31.6%)	45 (39.1%)	0.267*	77 (26.2%)	41 (26.8%)	0.980*
	Prior stroke, n (%)		19 (13.7%)	11 (9.6%)	0.416*	46 (15.5%)	19 (12.3%)	0.431*
**Pharmacotherapy**								
	Beta-blockers, n (%)		130 (93.5%)	108 (93.9%)	1.000*	282 (94.9%)	146 (94.2%)	0.905*
	SGLT2 inhibitors, n (%)		46 (33.1%)	83 (72.2%)	<0.001*	94 (40.7%)	111 (71.6%)	<0.001*
	MRAs, n (%)		108 (77.7%)	104 (90.4%)	0.011*	241 (81.1%)	143 (92.3%)	0.003*
	Furosemide, n (%)		81 (58.3%)	62 (53.9%)	0.568*	127 (55.0%)	95 (61.3%)	0.261*
	Thiazides, n (%)		17 (12.2%)	7 (6.1%)	0.131	13 (5.6%)	7 (4.5%)	0.815†
	Calcium channel blockers, n (%)		22 (15.8%)	1 (0.9%)	<0.001†	13 (4.4%)	4 (2.6%)	0.440†
	Hydralazine, n (%)		12 (8.6%)	5 (4.3%)	0.212†	6 (2.6%)	6 (3.9%)	0.555†
	Nitrates, n (%)		17 (12.2%)	6 (5.2%)	0.077†	11 (3.7%)	6 (3.9%)	1.000†
	Amiodarone, n (%)		15 (10.8%)	24 (20.9%)	0.041*	52 (17.5%)	45 (29.0%)	0.007*
	Ivabradine, n (%)		6 (4.4%)	13 (11.3%)	0.054†	4 (1.4%)	12 (7.8%)	0.001†
	Digoxin, n (%)		8 (5.8%)	7 (6.1%)	1.000†	32 (10.8%)	19 (12.3%)	0.752*
	Aspirin, n (%)		72 (51.8%)	46 (40.0%)	0.080*	93 (31.4%)	47 (30.3%)	0.895*
	P2Y12 antagonists, n (%)		19 (13.7%)	13 (11.3%)	0.707*	24 (8.1%)	7 (4.5%)	0.174†
	Anticoagulants, n (%)		28 (20.1%)	35 (30.4%)	0.081*	105 (35.5%)	52 (33.8%)	0.798*
	Statins, n (%)		101 (72.7%)	85 (73.9%)	0.935*	193 (65.0%)	94 (60.6%)	0.420*
	Ezetimibe, n (%)		7 (5.1%)	10 (8.7%)	0.318†	13 (5.7%)	11 (7.2%)	0.719*
	Fibrates, n (%)		1 (0.7%)	4 (3.5%)	0.182†	5 (2.2%)	1 (0.7%)	0.408†
	Metformin, n (%)		47 (33.8%)	38 (33.0%)	1.000*	49 (16.5%)	21 (13.5%)	0.493*
	Sulfonylureas, n (%)		15 (10.8%)	8 (7.0%)	0.381†	14 (6.1%)	3 (1.9%)	0.074†
	Insulin, n (%)		25 (18.0%)	15 (13.0%)	0.366*	35 (11.8%)	14 (9.0%)	0.456*
**Laboratory parameters**								
	Creatinine (mg/dL), Mdn (IQR)		1.19 (0.98–1.51)	1.19 (0.98–1.51)	0.948§	1.13 (0.90–1.39)	1.12 (0.92–1.46)	0.447§
	GFR (mL/min), M ± SD		68.3 ± 20.4	71.8 ± 21.0	0.178‡	74.1 ± 22.7	73.1 ± 21.7	0.675‡
	Sodium (mEq/L), M ± SD		139.2 ± 5.2	140.4 ± 3.3	0.037‡	138.4 ± 4.3	139.6 ± 3.6	0.002‡
	Potassium (mEq/L), M ± SD		4.62 ± 0.54	4.64 ± 0.48	0.847‡	4.61 ± 0.60	4.55 ± 0.54	0.232‡
	Blood glucose (mg/dL), Mdn (IQR)		101.0 (89.0–118.0)	101.0 (89.5–129.5)	0.629§	98.5 (85.0–123.7)	95.0 (85.0–114.5)	0.256§
	Hemoglobin A1c (%), Mdn (IQR)		6.10 (5.62–7.15)	6.10 (5.60–7.20)	0.925§	6.0 (5.6–6.5)	5.90 (5.6–6.4)	0.374§
	Total cholesterol (mg/dL), M ± SD		166.7 ± 56.6	164.8 ± 51.8	0.781‡	168.5 ± 47.1	171.7 ± 50.0	0.509‡
	HDL cholesterol (mg/dL), M ± SD		96.0 ± 53.8	100.2 ± 39.4	0.480‡	104.3 ± 49.2	102.3 ± 42.2	0.650‡
	LDL cholesterol (mg/dL), M ± SD		43.9 ± 15.1	41.3 ± 12.7	0.141‡	45.2 ± 13.2	45.1 ± 15.2	0.985‡
	Triglycerides (mg/dL), Mdn (IQR)		133.0 (89.0–179.0)	129.0 (87.0–182.0)	0.993§	116.0 (78.0–175.0)	119.0 (81.0–165.0)	0.897§
	BNP (pg/mL), Mdn (IQR)		426.0 (258.0–680.0)	334.0 (143.5–1.771.5)	1.000§	242.7 (68.5–953.6)	199.0 (105.0–1.670.5)	0.579§
	AST (U/L), Mdn (IQR)		22.0 (18.0–29.0)	23.0 (17.0–29.0)	0.915§	25.0 (19.0–34.0)	24.0 (19.0–30.0)	0.337§
	ALT (U/L), Mdn (IQR)		21.0 (16.0–29.7)	22.0 (16.0–32.5)	0.706§	22.0 (15.0–34.0)	22.0 (16.0–31.0)	0.942§

* Continuity-corrected Pearson's chi-squared test

† Fisher's exact test

‡ variance-adjusted independent Student t test

§ Mann–Whitney test.

ALT: alanine aminotransferase: AST: aspartate aminotransferase; BMI: body mass index; BNP: B-type natriuretic peptide; COPD: chronic obstructive pulmonary disease; GFR: glomerular filtration rate; HDL: high-density lipoprotein; IQR: interquartile range; LDL: low-density lipoprotein; LVEF: left ventricular ejection fraction; M: mean; Mdn: median; MI: myocardial infarction; MRAs: mineralocorticoid receptor antagonists; SD: standard deviation; SGLT2: sodium-glucose linked transporter 2.

Regarding HF pharmacotherapy, the sacubitril/valsartan group more often received sodium-glucose cotransporter 2 (SGLT2) inhibitors and mineralocorticoid receptor antagonists (MRAs). These individuals were also less likely to receive calcium channel blockers (CCBs) and more likely to receive amiodarone (Table 1). No significant differences were observed for other drugs (beta-blockers, diuretics, vasodilators, other antiarrhythmics, antidiabetics, lipid-lowering, and antithrombotics). The only statistically significant difference regarding laboratory parameters was slightly higher natremia in the sacubitril/valsartan group (Table 1).

### Propensity score matching

Based on clinical knowledge and statistically significant differences in the descriptive analysis, the following covariate candidates were entered into LASSO regression: sex, age, body mass index (BMI), LVEF, glomerular filtration rate (GFR), classical cardiovascular risk factors (obesity, hypertension, type 2 diabetes mellitus, dyslipidemia, smoking history), the major HF etiologies (ischemic, hypertensive, Chagas cardiomyopathy, dilated cardiomyopathy, valvular), other HF drugs (beta-blockers, SGLT2 inhibitors, and MRAs), antiarrhythmics, diuretics, antidiabetics, lipid-lowering agents, antithrombotic agents, and metabolic laboratory parameters (hemoglobin A1c, lipid profile). The treatment variable was set as sacubitril/valsartan use, and the outcome variable as combined hospitalization or death.

LASSO cross-validation returned a minimum lambda of 0.052, with the following coefficients: LVEF, smoking history, SGLT2 inhibitor use, CCB use, and amiodarone use. Participants were matched based on their propensity score for the use of sacubitril/valsartan. The following covariates were used for the calculation of the propensity score: age, LVEF, smoking history, SGLT2 inhibitor use, MRA use, CCB use, and amiodarone use. The mean standardized difference for distance was 1.27 before matching, and 0.07 after matching (Supplementary table 1). The mean standardized difference for covariates ranged from −1.61 to 0.87 before matching, and from −0.07 to 0.07 after matching. Before matching, the population had 115 individuals treated with sacubitril/valsartan 97/103 mg twice daily and 139 controls (treated with either enalapril 20 mg twice daily or losartan 100 mg daily) free from missing data on covariates. The matching process led to a loss of 60 controls, but greatly increased comparability between groups.

### Outcomes

Over the median follow-up of 23.9 months (IQR 11.9–41.1), there were 70 primary endpoint events (56 first hospitalizations and 32 deaths). There was a nominal but not statistically significant reduction in combined hospitalization or death in the sacubitril/valsartan group (Table 2). Similar results were observed for hospitalization, all-cause mortality, and 3p-MACE (Table 2). The notable exception was in NYHA class progression, in which improvement was more frequent in the sacubitril/valsartan group (Table 2).

**Table 2:** Outcomes after propensity score matching for sacubitril/valsartan compared to either enalapril or losartan

Variable			Main analyses			Sensitivity analyses		
			Enalapril 20 mg twice daily or losartan 100 mg daily (n = 79)	Sacubitril/valsartan 97/103 mg twice daily (n = 115)	p-value	Submaximal enalapril or losartan (n=132)	Submaximal sacubitril/valsartan (n=155)	p-value
**Hospitalization or death, n (%)**			31 (39.7%)	39 (33.9%)	0.500*	45 (34.9%)	55 (35.9%)	0.951*
	HF hospitalization or death, n (%)		12 (16.0%)	19 (16.5%)	1.000*	24 (18.5%)	26 (17.1%)	0.888*
	At least 1 hospitalization, n (%)		24 (30.8%)	32 (27.8%)	0.779*	38 (29.2%)	41 (26.8%)	0.748*
	At least 2 hospitalizations, n (%)		5 (6.4%)	6 (5.2%)	0.759†	18 (13.8%)	12 (7.8%)	0.150*
	At least 3 hospitalizations, n (%)		2 (2.6%)	3 (2.6%)	1.000†	6 (4.6%)	8 (5.2%)	1.000†
	At least 4 hospitalizations, n (%)		1 (1.3%)	3 (2.6%)	0.649†	1 (0.8%)	3 (2.0%)	0.627†
	HF hospitalization, n (%)		9 (12.0%)	14 (12.2%)	1.000†	19 (14.6%)	18 (11.8%)	0.610*
**All-cause death, n (%)**			15 (19.2%)	17 (14.8%)	0.536*	31 (24%)	29 (19.0%)	0.373*
	CV death, n (%)		9 (12.2%)	15 (13.2%)	1.000†	23 (18%)	21 (14.2%)	0.490*
		MI death, n (%)	2 (2.7%)	2 (1.8%)	0.647†	1 (0.8%)	1 (0.7%)	1.000†
		Stroke death, n (%)	0 (0.0%)	1 (0.9%)	1.000†	1 (0.8%)	3 (2.0%)	0.626†
		HF death, n (%)	6 (8.1%)	9 (7.9%)	1.000†	18 (14.1%)	15 (10.1%)	0.414*
		Other CV cause death, n (%)	1 (1.4%)	3 (2.6%)	1.000†	3 (2.3%)	2 (1.4%)	0.666†
**Non-CV death, n (%)**			4 (5.4%)	3 (2.6%)	0.436†	9 (7.0%)	4 (2.7%)	0.152†
	Infectious cause death, n (%)		2 (2.7%)	2 (1.8%)	0.647†	5 (3.9%)	2 (1.4%)	0.256†
		COVID-19 death, n (%)	1 (1.4%)	0 (0.0%)	0.394†	3 (2.3%)	1 (0.7%)	0.340†
**3p-MACE, n (%)**			12 (16.2%)	17 (14.9%)	0.972*	28 (21.9%)	23 (15.5%)	0.231*
	Non-fatal MI, n (%)		2 (2.6%)	1 (0.9%)	0.567†	3 (2.3%)	3 (2.0%)	1.000†
	Non-fatal stroke, n (%)		2 (2.6%)	1 (0.9%)	0.567†	6 (4.6%)	4 (2.6%)	0.521†
NYHA class progression								
	No change, n (%)		44 (64.7%)	52 (48.1%)	0.046*	65 (55.1%)	88 (60.3%)	0.469*
	Improvement, n (%)		15 (22.1%)	46 (42.6%)	0.009*	34 (28.8%)	43 (29.5%)	1.000*
	Worsening, n (%)		9 (13.2%)	10 (9.3%)	0.459†	19 (16.1%)	15 (10.3%)	0.222*

* Continuity-corrected Pearson's chi-squared test

† Fisher's exact test.

CV: cardiovascular; HF: heart failure; MACE: major adverse cardiovascular event; MI: myocardial infarction; NYHA: New York Heart Association.

ATET analysis confirmed these findings, showing no significant reductions for the primary outcome, or its components (Table 3). It also revealed an even stronger association for NYHA class improvement (Table 3).

**Table 3:** Average treatment effect on the treated for sacubitril/valsartan compared to either enalapril or losartan (maximum dosages)

Variable		Main analyses				Sensitivity analyses			
		β	SE	p-value	OR (95% CI)	β	SE	p-value	OR (95% CI)
**Hospitalization or death**		−0.431	0.311	0.165*	0.650 (0.354–1.195)	0.202	0.271	0.455*	1.224 (0.702–2.081)
	HF hospitalization or death	−0.236	0.391	0.546*	0.790 (0.367–1.700)	0.025	0.336	0.941*	1.025 (0.531–1.980)
	At least 1 hospitalization	−0.286	0.324	0.377*	0.751 (0.398–1.418)	0.001	0.290	0.998*	1.001 (0.567–1.765)
	At least 2 hospitalizations	−0.254	0.628	0.686*	0.776 (0.227–2.657)	−0.667	0.399	0.094*	0.513 (0.235–1.121)
	HF hospitalization	−0.428	0.421	0.310*	0.652 (0.286–1.488)	−0.230	0.367	0.531*	0.795 (0.387–1.631)
**All-cause death**		−0.214	0.410	0.602*	0.808 (0.362–1.802)	−0.049	0.321	0.879*	0.952 (0.508–1.786)
	CV death	0.057	0.465	0.902*	1.059 (0.426–2.635)	−0.127	0.351	0.716*	0.880 (0.443–1.751)
	HF death	0.523	0.660	0.428*	1.688 (0.463–6.154)	−0.226	0.396	0.568*	0.797 (0.367–1.733)
**3p-MACE**		−0.193	0.415	0.642*	0.824 (0.365–1.861)	−0.268	0.328	0.415*	0.765 (0.402–1.456)
**NYHA class progression**									
	No change	−0.942	0.338	0.005*	0.390 (0.201–0.757)	0.153	0.266	0.564*	1.165 (0.692–1.962)
	Improvement	1.362	0.411	0.001*	3.902 (1.745–8.726)	0.212	0.300	0.480*	1.236 (0.687–2.223)
	Worsening	−0.427	0.491	0.385*	0.653 (0.249–1.710)	−0.660	0.367	0.072*	0.517 (0.252–1.061)

* Weighted logistic regression and log odds (beta).

CI: confidence interval; CV: cardiovascular; HF: heart failure; NYHA: New York Heart Association; OR: odds ratio; SE: standard error; 3p-MACE: 3-point major adverse cardiovascular events.

### Sensitivity analysis

We also examined data from the 452 patients receiving any non-maximal dosages of the investigated drugs. This population was 61.5% male, with a mean age of 61.4 years and a median follow-up of 19.3 (IQR 8.7–33.7) months. A total of 155 patients (34.2%) used sacubitril/valsartan, 201 (44.4%) enalapril, and 96 (21.2%) losartan. Differences in population characteristics stratified by drug were similar to those seen with optimal dosages, with a lower LVEF and more frequent use of SGLT2 inhibitors, MRAs, and amiodarone in the sacubitril/valsartan group. After PSM (Supplementary Table 2), no differences were observed for the primary or secondary outcomes, which was confirmed by ATET.

## Discussion

This study used real-world data from an observational cohort to compare clinical outcomes of patients with HFrEF prescribed the guideline-recommended maximum dosages of sacubitril/valsartan (97/103 mg twice daily) versus either enalapril (20 mg twice daily) or losartan (100 mg daily). No significant reductions were observed in the primary outcome, combined hospitalization or death (odds ratio [OR] 0.650, 95% CI 0.354–1.195, p = 0.165), nor in its components, first hospitalization (OR 0.751, 95% CI 0.398–1.418, p = 0.377) and all-cause mortality (OR 0.808, 95% CI 0.362–1.802, p = 0.602). Our findings did not support reductions in hospitalization or mortality for sacubitril/valsartan compared to enalapril or losartan at guideline-recommended maximum dosages.

Following the PARADIGM-HF trial, subsequent studies comparing sacubitril/valsartan with equivalent dosages of ACE inhibitors or ARBs found no statistically significant differences between groups in patients with either preserved or reduced LVEF.^11-13^ In the LIFE trial, among patients with advanced HFrEF, sacubitril/valsartan failed to further reduce NT-proBNP compared to valsartan (area under the curve 0.95, 95% CI 0.84–1.08, p = 0.45).^11^ Similarly, the PARADISE-MI trial, which enrolled patients with left ventricular dysfunction following myocardial infarction, did not demonstrate superiority of sacubitril/valsartan over ramipril in preventing cardiovascular death or HF events (hazard ratio 0.90, 95% CI 0.78–1.04, p = 0.17).^12^ Furthermore, a recent meta-analysis compared sacubitril/valsartan with ACE inhibitors and ARBs at equivalent dosages and found no significant reductions in all-cause mortality (risk ratio 0.90, 95% CI 0.76–1.07, p = 0.38, I² = 0%).^13^ These studies also support the hypothesis that the favorable outcomes of sacubitril/valsartan in the PARADIGM-HF trial may have been influenced, at least partially, by the use of a suboptimal control regimen.

Sacubitril/valsartan was strongly associated with improvement in NYHA functional class (OR 3.902, 95% CI 1.745–8.726, p = 0.001), even in the absence of statistically significant reductions in hospitalization or mortality. This finding may be related to the enhanced natriuretic and hemodynamic effects of neprilysin inhibition. It is noteworthy that no significant differences were observed in diuretic use between the groups, and that PSM achieved near-perfect balance for SGLT2 inhibitors (72.1% vs. 70.0%) and MRAs (90.4% vs. 91.1%). Although covariate balance improved after matching, residual confounding cannot be fully excluded. This hypothesis was further tested through multivariate logistic regression (accounting for age, sex, LVEF, GFR, HF drugs, and diuretics), which showed similar results (sacubitril/valsartan with an OR of 2.805, 95% CI 1.275–6.172, p = 0.010) (Supplementary Table 3). This is consistent with other studies showing improvement in NYHA class and quality of life scores, despite no significant reductions in hospitalization or mortality.^4,13^ However, this finding was not observed in the sensitivity analysis, possibly reflecting greater heterogeneity in treatment exposure, as well as the subjective nature of NYHA classification.

A final aspect worth mentioning is the economic context surrounding sacubitril/valsartan use. Considering guideline-recommended maximum dosages and retail prices identified online in United States pharmacies, sacubitril/valsartan costs approximately USD 600 per month, whereas enalapril and losartan are available at approximately USD 25 and 10 per month, respectively. In the study setting, despite the generic drug policy, which allows low-cost production of medications, sacubitril/valsartan remains considerably more expensive, costing approximately USD 55 per month, compared with enalapril and losartan, available for approximately USD 2 and 3 per month, respectively. No formal pharmacoeconomic or cost-effectiveness analyses were performed in this study. Therefore, these price differences should be interpreted only as contextual information. Further dedicated pharmacoeconomic studies are needed to evaluate the cost-effectiveness of sacubitril/valsartan across different healthcare settings.

The findings of this study underscore the importance of incorporating real-world evidence into the evaluation of therapeutic strategies. In an increasingly complex landscape marked by multiple pharmacological options, understanding how new therapies perform outside the controlled environment of clinical trials is essential. Sacubitril/valsartan should be assessed not only for efficacy, but also in terms of applicability, affordability, and implementation across different healthcare contexts, particularly in low- and middle-income countries with constrained healthcare resources. Although our findings provide relevant real-world insights, direct comparisons with randomized clinical trial outcomes should be interpreted cautiously given the observational nature of the present study.

### Limitations

The main limitation of our study is its relatively small sample size. The nominal reduction in the primary outcome observed in the sacubitril/valsartan group raises the question of whether this difference would reach statistical significance in a larger cohort. Using GPower 3.1.9.7, the statistical power (1 − β error) for the measured outcomes was excellent (0.893). This suggests that our sample size was sufficient to detect the observed effect, although the reduction in effective sample size after PSM may have limited the detection of smaller differences between groups.

The observational and partially retrospective design is another limitation. To account for baseline differences between treatment groups, we performed PSM. Without this adjustment, the higher prevalence of SGLT2 inhibitors (72.2% vs. 33.1%, p < 0.001) and MRAs (90.4% vs. 77.7%, p = 0.011) in the sacubitril/valsartan group could have substantially influenced outcomes. Although covariate balance improved after matching, residual confounding cannot be fully excluded. Furthermore, the exclusion of patients due to missing data and PSM may have introduced selection bias and should be considered when interpreting the findings.

Data collection also presents limitations inherent to the study design. Treatment adherence was not systematically assessed, particularly in the retrospective portion of the cohort, which may have influenced the observed outcomes. Likewise, outcome ascertainment was primarily based on medical records, with partial prospective follow-up, which may have introduced variability in data collection.

Methodological considerations should also be acknowledged. Combining enalapril and losartan into a single comparator group may have introduced pharmacological heterogeneity, although this approach was intended to reflect conventional renin–angiotensin system blockade strategies in routine clinical practice. In addition, the regression models used evaluated cumulative event occurrence and did not account for time-to-event differences, which may limit assessment of temporal outcome patterns.

The long follow-up period was overall positive as it allowed for more endpoint events to happen. However, the start of the observation period in August 2010 creates a potential bias, as the sacubitril/valsartan group would be more recent, having better access to modern therapies for HF (i.e., SGLT2 inhibitors) and other diseases. This problem was partially addressed by PSM. To further isolate the impact of sacubitril/valsartan, we also estimated the ATET. Although these approaches reduce confounding, residual temporal bias cannot be fully excluded.

## Conclusion

In this real-world study of patients with HFrEF, treatment with sacubitril/valsartan was not associated with statistically significant reductions in the combined outcome of hospitalization or death when compared with guideline-recommended maximum dosages of enalapril or losartan. Similarly, no statistically significant differences were observed for the individual outcomes of hospitalization, all-cause mortality, or major adverse cardiovascular events (3p-MACE).

Despite the lack of impact on these key clinical events, treatment with sacubitril/valsartan was strongly associated with an improvement in patients’ functional status, as assessed by the NYHA classification.

These findings indicate that, in a real-world setting where background therapies are optimized, the magnitude of benefit of sacubitril/valsartan on mortality and hospitalization may differ from that observed in randomized clinical trials. The results highlight the need for further research to clarify the role of sacubitril/valsartan in specific patient subgroups, including dedicated studies designed to evaluate cost-effectiveness across different healthcare contexts.

## Data Availability

The underlying content of the research text is contained within the manuscript.

## *Supplemental Materials

Supplemental Materials For additional information, please click here.
